# A New Approach to Vascular Screening: Identification of Impaired Vascular Function Using the FMSF Technique

**DOI:** 10.3390/s24061721

**Published:** 2024-03-07

**Authors:** Andrzej Marcinek, Joanna Katarzynska, Jerzy Gebicki

**Affiliations:** 1 Institute of Applied Radiation Chemistry, Lodz University of Technology, 90-924 Lodz, Poland; andrzej.marcinek@p.lodz.pl; 2 Angionica Ltd., 90-924 Lodz, Poland; joanna.katarzynska@angionica.com.pl

**Keywords:** FMSF technique, NADH fluorescence, vascular circulation, vascular screening

## Abstract

Arterial blood pressure monitoring plays an important role in preventive medicine, allowing, in selected cases, the identification of vascular dysfunction. In this review, we propose a new non-invasive approach to assessment of the circulatory system, based on its reaction to hypoxia induced by post-occlusive reactive hyperemia (PORH). Three key parameters can be used for vascular screening: the Reactive Hyperemia Response (RHR), which represents the overall reaction of the macro- and microcirculation to transient hypoxia; Hypoxia Sensitivity (HS), which reflects hypoxia-induced activation of myogenic oscillations of the microcirculation; and Normoxia Oscillatory Index (NOI), which characterizes microcirculatory oscillations under normoxia conditions. A method for assessing these parameters, analogous in simplicity to arterial blood pressure measurement, is provided by the Flow Mediated Skin Fluorescence (FMSF) technique. Reference values are proposed based on numerous test measurements.

## 1. Introduction

The diagnosis and treatment of vascular diseases incurs tremendous costs to the healthcare system. Early diagnosis of vascular dysfunction is not always possible, as it is often not manifested by characteristic symptoms. Hypertension is a common example of such silent vascular dysfunction. Therefore, occasional monitoring of arterial blood pressure is recommended as a preventative measure. However, blood pressure monitoring is only able to identify selected cases of vascular dysfunction. Many other cases of silent vascular dysfunction are not manifested by changes in blood pressure.

Vascular system dysfunction often results in insufficient delivery of oxygen and nutrients to tissues, due to hypoxia. There are techniques available for the assessment of the body’s reaction to hypoxia. The classical technique, called flow-mediated dilation (FMD) and regarded as the gold standard [1,2,3], is based on stimulation of the vascular circulation in response to the post-occlusive reactive hyperemia (PORH) [4]. However, FMD only measures the reaction of the macrocirculation to transient hypoxia. Despite great research interest in the use of the FMD technique, its widespread adoption as a routine diagnostic technique has been hindered by the technical complexities involved in its execution.

Changes in the functioning of the endothelium of large blood vessels are often preceded by dysfunction of vascular microcirculation [5]. Because cutaneous microcirculation is representative for the assessment of systemic microcirculation, its dysfunctions and pathologies [6,7,8] and is readily accessible for monitoring, changes in epidermal cell functioning in response to ischemia are a sensitive marker of early vascular circulation disorders.

In light of this, there is growing demand for a diagnostic tool that is both simple and non-invasive, for assessment of the body’s reaction to transient hypoxia. Addressing this need is the Flow Mediated Skin Fluorescence (FMSF) technique [9,10,11,12,13,14,15,16,17]. The FMSF technique stands out for its ability to provide information on distinguishable macro- and microcirculatory responses to transient hypoxia.

The FMSF technique is based on the measurement of the strongest fluorescence emitted from human skin, nicotinamide adenine dinucleotide (NADH) fluorescence. A significant part of the exciting light is absorbed by the epidermis and papillary dermis [11,18,19,20]; hence, the emission of NADH and its changes are related to changes in the mitochondrial redox balance of the NADH/NAD^+^ pair in epidermal cells, which are the final recipients of oxygen from the circulatory system.

NADH fluorescence has previously been used to determine mitochondrial function in vivo [21,22,23,24]. Because only the reduced form of the NADH/NAD+ pair fluoresces, limited information can be obtained regarding the redox balance of this coenzyme as an indicator of mitochondrial function. However, the reduced form of the coenzyme (NADH) accumulates under ischemia and hypoxia, while it is oxidized during hyperemia. Hence, changes observed during the PORH test can be used to assess the NADH/NAD^+^ balance in epidermal cells and vascular response to ischemic conditions.

In this review, we summarize the major conclusions from numerous research studies using the FMSF technique for non-invasive assessment of vascular circulation. Drawing from data collected from the hundreds of test subjects, both healthy individuals and those with diagnosed vascular diseases, our focus is on identifying cases exhibiting impaired vascular function. We are convinced that the FMSF technique holds significant promise for use in vascular screening, providing a powerful diagnostic tool for characterization of vascular circulation.

## 2. Brief Description of the FMSF Methodology

**Figure 1:**
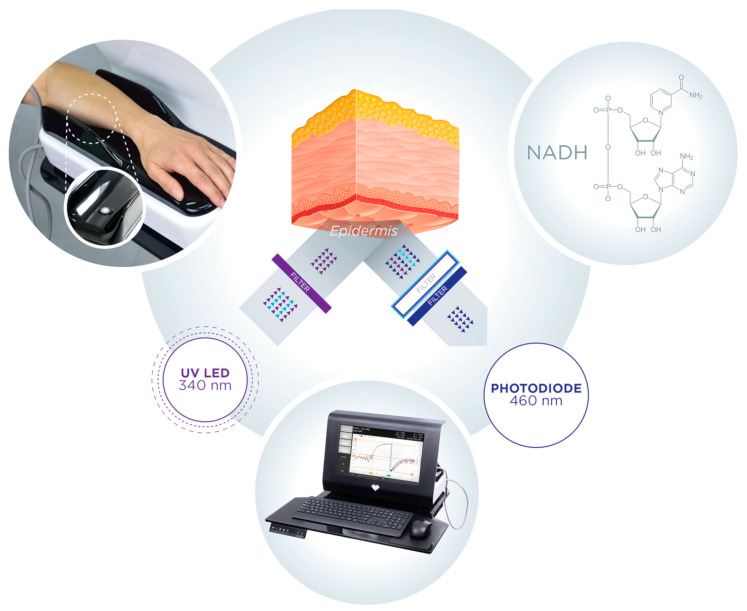
Operating principle of AngioExpert.

The Flow Mediated Skin Fluorescence (FMSF) technique measures changes in the in-tensity of nicotinamide adenine dinucleotide (NADH) fluorescence from the skin on the forearm (Figure 1). The changes are stimulated by blocking and releasing blood flow in the brachial artery. The skin is characterized by a specific metabolism. The epidermal layer of skin is not directly vascularized, and oxygen and nutrients are transported from the dermis by diffusion. Therefore, epidermal cell metabolism can be considered a unique and sensitive marker of early dysfunction in vascular circulation and metabolic regulation.

**Figure 2:**
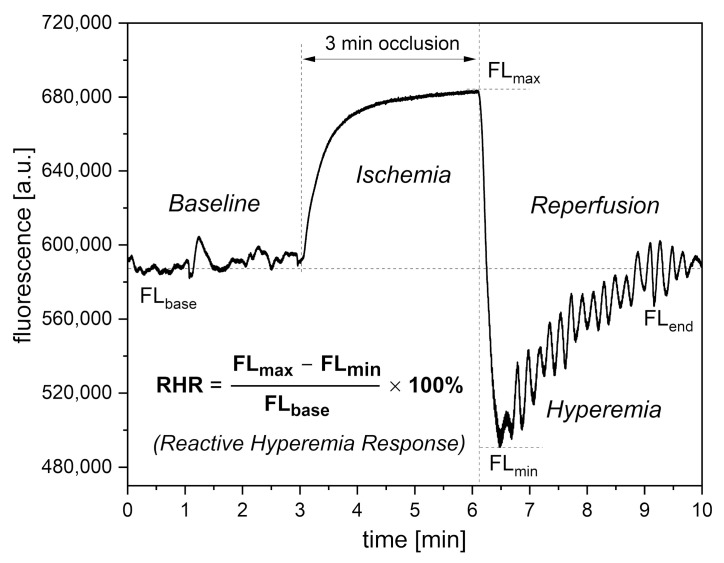
Definition of the RHR parameter.

AngioExpert is a diagnostic tool based on the FMSF technique, produced by Angionica Ltd. (Lodz, Poland). The measurement protocol has been described in detail elsewhere [12,17]. Figure 2 shows an exemplary FMSF trace, consisting of three parts: the baseline, the ischemic response (IR), and the hyperemic (reperfusion) response (HR).

In the PORH test, an increase in NADH fluorescence is observed during the ischemic response associated with brachial artery occlusion. After 3 min, the occlusion is released and NADH fluorescence drops below the baseline, reaching minimum (hyperemia, 20–30 s), followed by a return to baseline values (reperfusion, approximately 3 min).

On the baseline and reperfusion lines, microcirculation oscillations known as flowmotion are clearly visible [25,26]. These microcirculatory oscillations in the low-frequency range (<0.15 Hz) fit into several periodic activities, classified as endothelial (<0.021 Hz), neurogenic (0.021–0.052 Hz), and myogenic (0.052–0.15 Hz). Characteristic parameters have been defined for each part of the FMSF trace and can be used for precise analysis of the vascular function, as discussed in previous studies [27,28,29]. Based on extensive experience examining hundreds of cases, we decided to select only three key parameters for vascular screening purposes: Reactive Hyperemia Response (RHR), Hypoxia Sensitivity (HS), and Normoxia Oscillatory Index (NOI). These parameters will be presented in the subsequent sections.

## 3. Interpretation of Key FMSF Parameters for Vascular Screening

Of the three parameters selected for vascular screening, the RHR and HS parameters are particularly useful. The RHR parameter is defined in Figure 2. RHR is a unique parameter based on the combined responses from both the ischemic and hyperemic parts of the measured FMSF trace (RHR = IR_max_ + HR_max_). It represents the overall function of the vascular system, including both macro- and microcirculation [14,17]. The HS parameter represents a direct measure of the intensity of microcirculatory oscillations related to myogenic oscillations with frequencies in the range of 0.052–0.15 Hz, recorded during reperfusion [13,14,17]. Myogenic microcirculatory oscillations are a very sensitive measure of the microcirculatory response to hypoxia and can be monitored with high precision using the FMSF technique. As the values of the HS parameter can vary within a quite broad range, it is more practical to use a normally distributed log (HS).

**Figure 3:**
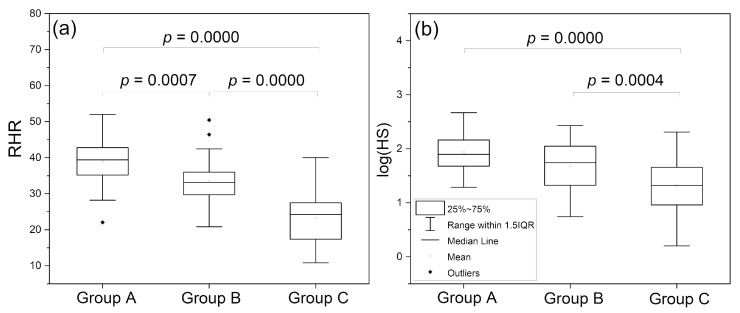
Comparison of RHR (**a**) and log (HS) (**b**) parameters in groups A, B, and C: A—highly trained endurance athletes, n = 50 (33 male, 17 female), mean age 22.0 (16–35 y.); B—healthy middle-aged individuals, n = 32 (19 male, 13 female), mean age 38.2 (30–50 y.); C—diabetes type 2 patients, n = 70 (40 male, 32 female), mean age 63.1 (45–80 y.). Reproduced from [14]. 2022, Dove Medical Press Ltd. Publisher.

**Figure 4:**
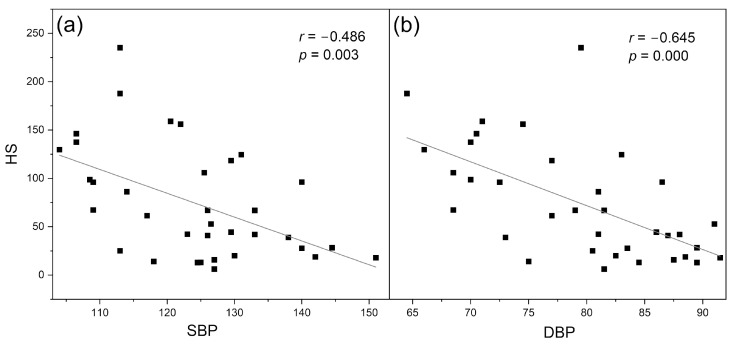
Correlation between the HS parameter and systolic (SBP) (**a**) and diastolic (DBP) (**b**) blood pressure for the group of healthy middle-aged individuals, n = 35 (22 males, 13 females), mean age 38.5 (30–50 y.). Reproduced from [31]. 2021, Elsevier B.V. RELX Group.

As can be seen in Figure 3, the parameters RHR and log (HS) nicely differentiate the groups of endurance athletes, healthy middle-aged individuals, and the patients with type 2 diabetes [14]. Both these parameters seem to be mutually correlated (*r* = 0.413, *p* = 0.0001). The RHR parameter can be used to characterize vascular health in a broad segment of the population and can be regarded as a vitality measure [30]. As shown in Figure 4, the HS parameter, representing microcirculatory oscillations in response to hypoxia, correlates with both systolic and diastolic blood pressure in healthy individuals [31]. It should potentially be taken into consideration in the diagnosis of arterial hypertension.

The baseline microcirculatory oscillations hold interesting diagnostic potential. The intensity and relative distribution of these oscillations in the low frequency range (<0.15 Hz) have been found to be sensitive to various stress factors. These include emotional stress, physical exhaustion, post-infection states, and hormonal deficiencies. The NOI parameter can be used for universal characterization of stress of various origins. The NOI parameter represents the contribution of endothelial and neurogenic oscillations relative to all oscillations detected at low-frequency intervals (<0.15 Hz). For emotional stress, physical exhaustion, or post-infection stress, the NOI parameters have values below 60%. In the case of hormonal deficiency frequently associated with erectile dysfunction, the NOI parameter values are in the range of 90–100% [15,17].

**Figure 5:**
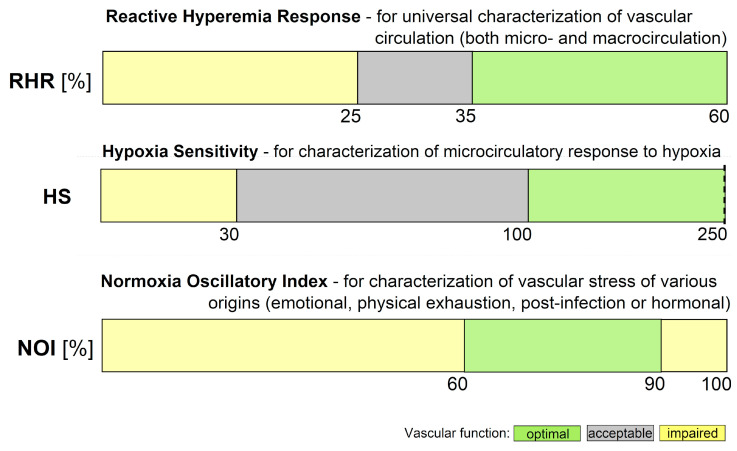
Ranges of key FMSF parameters (RHR—Reactive Hyperemia Response, HS—Hypoxia Sensitivity, NOI—Normoxia Oscillatory Index).

Based on our extensive experience, the function of the RHR, HS, and NOI parameters can be presented in the form of a colored strip, as shown in Figure 5. The most optimal values are shown in green and values indicating potential vascular dysfunction are shown in yellow. In the following section, we will present examples of cases with optimal or dysfunctional vascular circulation, to prove that the FMSF technique is indeed suitable for vascular screening.

## 4. Selected Examples of FMSF Measurements Indicating Impaired Vascular Function

### 4.1. Identification of Cases with Dysfunctional Vascular Circulation

**Figure 6:**
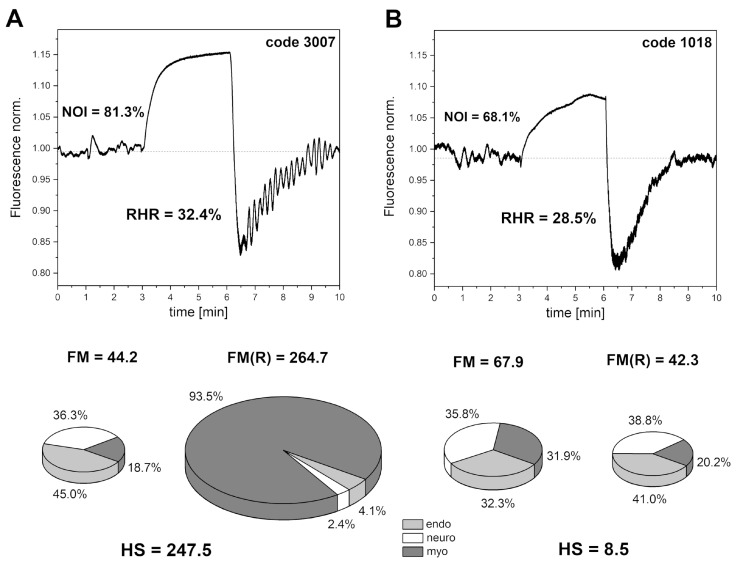
Exemplary FMSF traces recorded for (**A**) a patient with high flowmotion response to hypoxia (male, age 30 y.), and (**B**) a patient with low flowmotion response to hypoxia (male, age 34 y.). The calculated flowmotion parameters are shown below the traces. Reproduced from [12]. 2020, Frontiers Media S.A.

Figure 6 shows exemplary FMSF traces collected for two healthy individuals (A—male, age 30 y. and B—male, age 34 y.). There is a striking difference between the two recorded FMSF traces. In case B, the microcirculatory oscillations seen on the reperfusion line are very weak and the HS parameter has a value of 8.5. This observation clearly indicates that the microcirculatory reaction to hypoxia is impaired and myogenic oscillations are not effectively generated. In contrast, in case A, the measured HS parameter is very high (HS = 247.5), indicating a very good reaction of the microcirculation to hypoxia. The RHR and NOI parameters in each case lie in similar ranges. The observed impaired reaction of microcirculation to hypoxia in case B suggests dysfunctional signaling of the hypoxia-inducible factor (HIF), resulting in insufficient stabilization of HIF-1α [32,33]. As shown in Figure 4 [31], the HS parameter correlates with blood pressure and both of these variables seem to be regulated by HIF-1α in vascular smooth muscle cells [34,35]. Such dysfunction can appear in genetically predisposed individuals. Individuals with very low values for the HS parameter may have a predisposition towards arterial hypertension, poor tolerance of physical exercise, or poor tolerance to high altitude [36].

**Figure 7:**
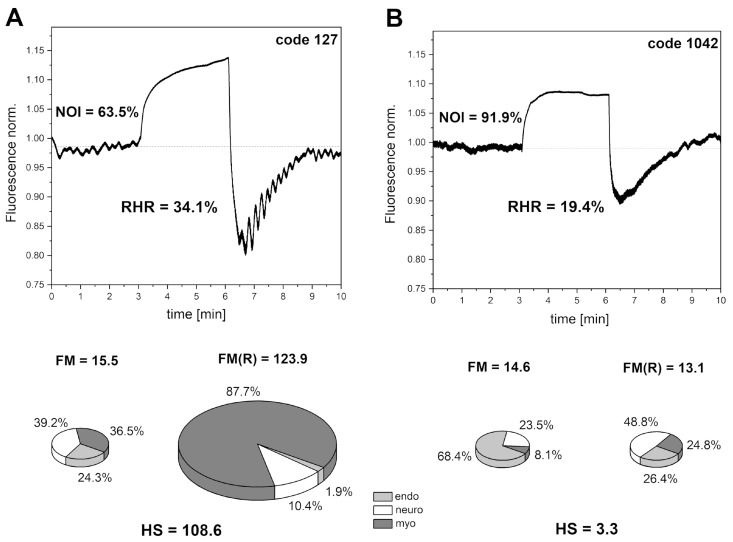
Exemplary FMSF traces recorded for (**A**) DM2 patient (female, age 70 y., disease duration 20 y., no vascular complications, and (**B**) DM2 patient (female, age 59 y., disease duration 18 y., diabetic foot). The calculated flowmotion parameters are shown below the traces.

Figure 7 shows exemplary FMSF traces for two patients with type 2 diabetes (DM2) (A—female, age 70 y., disease duration 20 y., no vascular complications and B—female, age 59 y., disease duration 18 y., diabetic foot). Notable differences are visible between the two cases. All FMSF parameters measured for patient A are optimal and no vascular complications are indicated, despite a long disease duration. In fact, we have observed that about 8–10% of DM2 patients studied have optimal FMSF parameters, typical for healthy individuals at a similar age [37]. FMSF is a very appropriate technique for identifying such patients. In contrast, patient B has much worse key FMSF parameters. The RHR is low, indicating impaired vascular function. The most pronounced observation concerns the HS parameter, which is very low (HS = 3.3). We have shown that such low values for the HS parameter are indicative of microvascular complications in diabetes [17,38]. In patients with diabetic foot ulcer (including patient B), a very low value of the HS parameter predicts a low prognosis for healing [39,40]. The FMSF technique has a unique diagnostic potential for assessing vascular function in diabetic patients, particularly those with microvascular complications.

**Figure 8:**
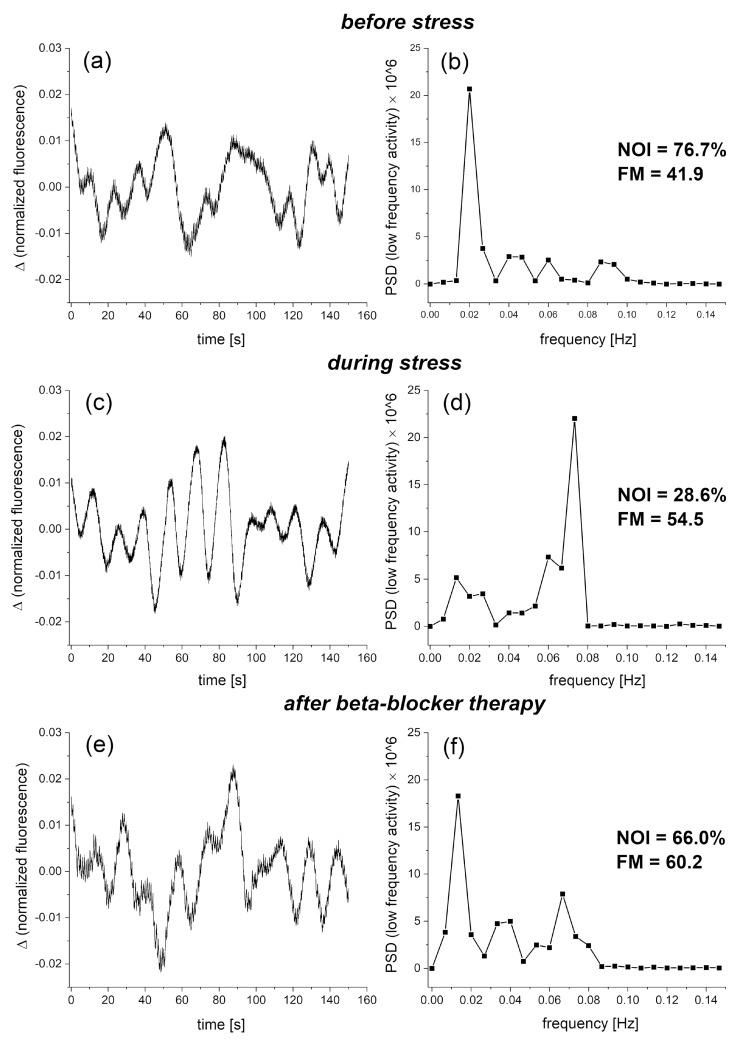
Exemplary FMSF baseline traces recorded for a prediabetes patient (male, age range 70–75 y). Changes in the fluorescence signal relative to the normalized baseline (left) and the corresponding Power Spectral Density (PSD) of the fluorescence signal in the intervals of endothelial (<0.021 Hz), neurogenic (0.021–0.052 Hz), and myogenic (0.052–0.15 Hz) activity (right): (**a**,**b**) changes recorded before the appearance of psychological stress; (**c**,**d**) changes observed under prolonged psychological stress; and (**e**,**f**) changes observed after a week of therapy with a beta-blocker (nebivolol at a daily dose of 1.25 mg). Reproduced from [15]. 2023, Dove Medical Press Ltd. Publisher.

It has long been recognized that prolonged psychological stress is an important risk factor for the entire vascular system [41]. Psychological stress is associated with increased levels of norepinephrine in the circulation, which can cause microvascular vasoconstriction. We have recently shown the effect of psychological stress on the microcirculation, using the FMSF technique [15]. Figure 8 presents exemplary FMSF baseline traces recorded for the studied patient before stress (segment a), during stress (segment c) and after therapy (segment e). The NOI parameter dropped from 76.7% before stress to 28.6% during psychological stress. Evidently, norepinephrine associated with psychological stress caused microvascular vasoconstriction, resulting in increased myogenic microcirculatory oscillations and as a consequence, a drop in the NOI parameter. After treatment with a low dose of beta-blocker, the NOI parameter returned to a normal value of 66.0%, similar to that before stress. The results presented in Figure 8 clearly indicate that the NOI parameter is suitable for evaluation of psychological stress, and that a low dose of beta-blocker can be recommended for mild psychological stress. It has been suggested previously that beta-adrenergic blockade attenuates negative, high arousal emotions in response to psychological stressor [42]. The use of the FMSF technique to assess the vascular consequences of psychological stress is a particularly attractive option, due to the non-invasive nature of the FMSF technique and the possible adaptation of this methodology for use in wearable devices.

### 4.2. Selection of Cases with No Contradictions for Amateur Physical Activity

**Figure 9:**
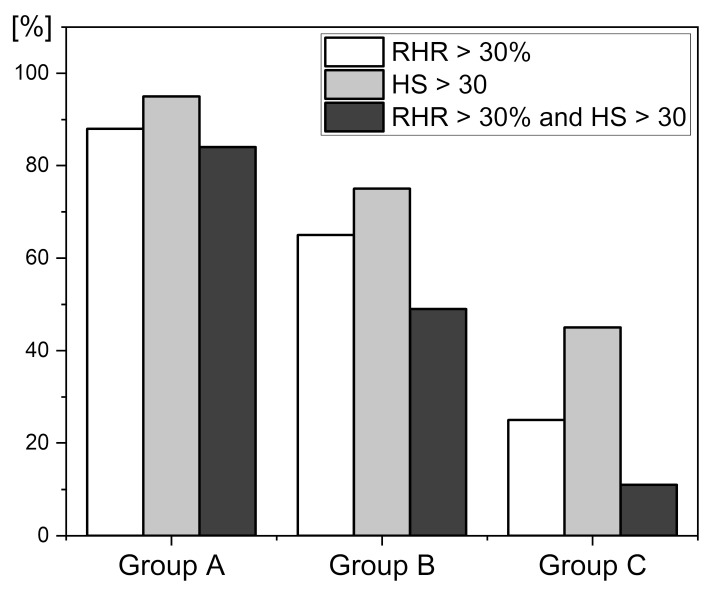
Distribution of the RHR and HS parameters in groups A, B, and C: A—highly trained athletes, n = 33, mean age 22.5 (16–35 y.), B—healthy middle-aged individuals, n = 35, mean age 38.5 (30–50 y.), C—diabetes type 2 patients, n = 72, mean age 62.7 (50–80 y.).

It is well known that physical exercise should be adequately planned in order to be safe. Physical exercise that is not rationally planned can negatively affect the vascular system, leading to the development of serious health problems. A simple, non-invasive diagnostic tool is needed to verify vascular health prior to physical activity. The FMSF technique admirably meets this requirement. After extensive studies involving professional and amateur athletes, we have selected ranges for the RHR and HS parameters that indicate safe levels for physical activity [13,14,43]. Within these ranges (RHR > 30% and HS > 30), there is low risk of counterindications for amateur physical activity. Figure 9 presents the distribution of the RHR, and HS (RHR > 30% and HS > 30) parameters for three groups: A—highly trained athletes, B—middle-aged individuals, and C—type 2 diabetes patients. The established precondition for amateur physical activity is fulfilled in about 85%, 50%, and 10% of individuals in groups A, B, and C, respectively. This result is worthy of special consideration. Only about 50% of healthy middle-aged individuals have an adequate vascular system prepared for amateur physical activity. We believe that such information should be seriously taken into consideration by those planning amateur physical activity. Based on our studies, the FMSF technique has unique diagnostic potential for assessing vascular health before undertaking physical activity. We and others have shown that regular training can improve these parameters, particularly the RHR parameter, which can be considered as the most important.

### 4.3. Presentation of Cases with Atypical Ischemic Response

**Figure 10:**
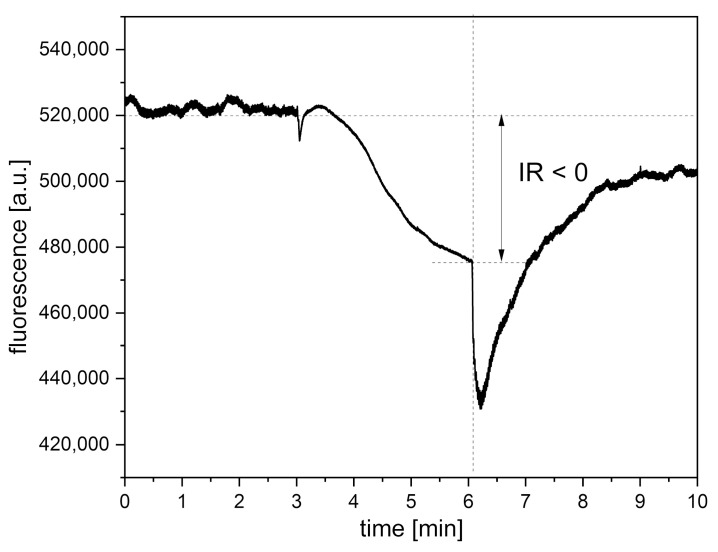
An exemplary FMSF trace with a negative (atypical) ischemic response (IR < 0) recorded for a cardiac patient (male, 71 y., ischemic heart disease, hypertension).

An observational study of patients with vascular diseases (n = 482) using the FMSF technique identified a sizable group of patients with a negative (atypical) ischemic response (n = 162) [30]. A similar observation was made in a study of hypertensive patients [44,45]. In both of these studies, about a third of the patients, mostly females, showed an atypical ischemic response, as discussed in [30,44]. In the case of healthy individuals, no such deviation in the ischemic response is seen. An exemplary FMSF trace recorded for a patient with a negative ischemic response is shown in Figure 10. It is clear that a negative ischemic response is associated with more severe clinical cases, regardless of the vascular problem analyzed. For example, the group with typical FMSF traces had on average HS = 15.5, while the HS value observed for the group with a negative ischemic response was 8.1 [30]. Cases with a negative ischemic response are very easy to identify, and they should be given special medical attention. Our experience suggests that in the case of females such deviations in the observed FMSF traces may also be linked to a hormonal disturbance [46].

## 5. Conclusions

Extensive research, encompassing both healthy subjects and patients with a range of vascular issues, has consistently demonstrated the efficacy of the Flow Mediated Skin Fluorescence (FMSF) technique in identifying cases of impaired vascular function, including many cases of silent vascular dysfunction that are not manifested by changes in blood pressure. The FMSF technique is able to distinguish macro- and microcirculatory responses to transient hypoxia. The risk of vascular complications has been found to be reduced when the RHR, HS, and NOI parameters are not significantly below the average values, especially if they simultaneously meet the conditions RHR > 30%, HS > 30, and NOI > 60%. The FMSF technique can therefore provide a powerful yet simple and non-invasive diagnostic tool for characterization of vascular circulation, with a wide variety of potential uses in preventative medicine.

## Data Availability

Not applicable.
